# Third‐trimester ultrasound for antenatal diagnosis of placenta accreta spectrum in women with placenta previa: results from the ADoPAD study

**DOI:** 10.1002/uog.24889

**Published:** 2022-09-01

**Authors:** N. Fratelli, F. Prefumo, C. Maggi, C. Cavalli, A. Sciarrone, A. Garofalo, E. Viora, P. Vergani, S. Ornaghi, M. Betti, I. Vaglio Tessitore, A. F. Cavaliere, S. Buongiorno, A. Vidiri, E. Fabbri, E. Ferrazzi, V. Maggi, I. Cetin, T. Frusca, T. Ghi, C. Kaihura, E. Di Pasquo, T. Stampalija, C. Belcaro, M. Quadrifoglio, M. Veneziano, F. Mecacci, S. Simeone, A. Locatelli, S. Consonni, N. Chianchiano, F. Labate, A. Cromi, E. Bertucci, F. Facchinetti, A. Fichera, D. Granata, F. D'Antonio, F. Foti, L. Avagliano, G. P. Bulfamante, G. Calì, the ADoPAD (Antenatal Diagnosis of Placental Adhesion Disorders) Working Group

**Affiliations:** ^1^ Division of Obstetrics and Gynecology, ASST Spedali Civili, Department of Clinical and Experimental Sciences University of Brescia Brescia Italy; ^2^ Obstetrics–Gynecological Ultrasound and Prenatal Diagnosis Unit, Department of Obstetrics and Gynecology Città della Salute e della Scienza Turin Italy; ^3^ University of Milan‐Bicocca, School of Medicine and Surgery, Department of Obstetrics and Gynecology Fondazione MBBM Onlus, San Gerardo Hospital Monza Italy; ^4^ Obstetrics and Gynaecology Unit, A. Manzoni Hospital, ASST Lecco Lecco Italy; ^5^ Dipartimento Scienze della Salute della Donna e del Bambino e di Sanità Pubblica Fondazione Policlinico Universitario ‘A. Gemelli’ IRCCS‐Università Cattolica del Sacro Cuore Rome Italy; ^6^ Obstetrics and Gynecology Unit Buzzi Children's Hospital, University of Milan Milan Italy; ^7^ Fondazione IRCCS Ca Granda Ospedale Maggiore Policlinico Milano, Unit of Obstetrics Milan Italy; ^8^ Department of Clinical and Community Sciences University of Milan Milan Italy; ^9^ Department of Medicine and Surgery, Obstetrics and Gynaecology Unit University of Parma Parma Italy; ^10^ Department of Medicine and Surgery University of Parma Parma Italy; ^11^ Unit of Fetal Medicine and Prenatal Diagnosis Institute for Maternal and Child Health, IRCCS Burlo Garofolo Trieste Italy; ^12^ Department of Medical, Surgical and Health Science University of Trieste Trieste Italy; ^13^ Obstetrics and Gynecology Unit Bolzano Hospital Bolzano Italy; ^14^ Department of Woman and Child's Health Careggi University Hospital Florence Italy; ^15^ University of Milan‐Bicocca, School of Medicine and Surgery, Obstetrics and Gynecology Unit, Carate Brianza Hospital, ASST Brianza Carate Brianza Italy; ^16^ Obstetrics and Gynecology Unit, Carate Brianza Hospital, ASST Brianza Carate Brianza Italy; ^17^ Fetal Medicine Unit, Bucchieri La Ferla–Fatebenefratelli Hospital Palermo Italy; ^18^ Department of Obstetrics and Gynaecology Azienda Ospedaliera Villa Sofia Cervello Palermo Italy; ^19^ Department of Medicine and Surgery University of Insubria Varese Italy; ^20^ Obstetrics and Gynecology Unit, Department of Medical and Surgical Sciences for Children and Adults University of Modena and Reggio Emilia School of Medicine Modena Italy; ^21^ Obstetrics and Gynecology Unit Bolognini Hospital Seriate Italy; ^22^ Center for Fetal Care and High‐Risk Pregnancy, Department of Obstetrics and Gynecology University of Chieti Chieti Italy; ^23^ Obstetrics and Gynecology Unit, Civico Hospital of Partinico Palermo Italy; ^24^ Department of Health Sciences Università degli Studi di Milano Milan Italy; ^25^ Department of Obstetrics and Gynaecology Arnas Civico Hospital Palermo Italy

**Keywords:** Cesarean section, diagnosis, low‐lying placenta, placenta accreta spectrum, placenta previa, ultrasound

## Abstract

**Objective:**

To evaluate the performance of third‐trimester ultrasound for the diagnosis of clinically significant placenta accreta spectrum disorder (PAS) in women with low‐lying placenta or placenta previa.

**Methods:**

This was a prospective multicenter study of pregnant women aged ≥ 18 years who were diagnosed with low‐lying placenta (< 20 mm from the internal cervical os) or placenta previa (covering the internal cervical os) on ultrasound at ≥ 26 + 0 weeks' gestation, between October 2014 and January 2019. Ultrasound suspicion of PAS was raised in the presence of at least one of these signs on grayscale ultrasound: (1) obliteration of the hypoechogenic space between the uterus and the placenta; (2) interruption of the hyperechogenic interface between the uterine serosa and the bladder wall; (3) abnormal placental lacunae. Histopathological examinations were performed according to a predefined protocol, with pathologists blinded to the ultrasound findings. To assess the ability of ultrasound to detect clinically significant PAS, a composite outcome comprising the need for active management at delivery and histopathological confirmation of PAS was considered the reference standard. PAS was considered to be clinically significant if, in addition to histological confirmation, at least one of these procedures was carried out after delivery: use of hemostatic intrauterine balloon, compressive uterine suture, peripartum hysterectomy, uterine/hypogastric artery ligation or uterine artery embolization. The diagnostic performance of each ultrasound sign for clinically significant PAS was evaluated in all women and in the subgroup who had at least one previous Cesarean section and anterior placenta. Post‐test probability was assessed using Fagan nomograms.

**Results:**

A total of 568 women underwent transabdominal and transvaginal ultrasound examinations during the study period. Of these, 95 delivered in local hospitals, and placental pathology according to the study protocol was therefore not available. Among the 473 women for whom placental pathology was available, clinically significant PAS was diagnosed in 99 (21%), comprising 36 cases of placenta accreta, 19 of placenta increta and 44 of placenta percreta. The median gestational age at the time of ultrasound assessment was 31.4 (interquartile range, 28.6–34.4) weeks. A normal hypoechogenic space between the uterus and the placenta reduced the post‐test probability of clinically significant PAS from 21% to 5% in women with low‐lying placenta or placenta previa in the third trimester of pregnancy and from 62% to 9% in the subgroup with previous Cesarean section and anterior placenta. The absence of placental lacunae reduced the post‐test probability of clinically significant PAS from 21% to 9% in women with low‐lying placenta or placenta previa in the third trimester of pregnancy and from 62% to 36% in the subgroup with previous Cesarean section and anterior placenta. When abnormal placental lacunae were seen on ultrasound, the post‐test probability of clinically significant PAS increased from 21% to 59% in the whole cohort and from 62% to 78% in the subgroup with previous Cesarean section and anterior placenta. An interrupted hyperechogenic interface between the uterine serosa and bladder wall increased the post‐test probability for clinically significant PAS from 21% to 85% in women with low‐lying placenta or placenta previa and from 62% to 88% in the subgroup with previous Cesarean section and anterior placenta. When all three sonographic markers were present, the post‐test probability for clinically significant PAS increased from 21% to 89% in the whole cohort and from 62% to 92% in the subgroup with previous Cesarean section and anterior placenta.

**Conclusions:**

Grayscale ultrasound has good diagnostic performance to identify pregnancies at low risk of PAS in a high‐risk population of women with low‐lying placenta or placenta previa. Ultrasound may be safely used to guide management decisions and concentrate resources on patients with higher risk of clinically significant PAS. © 2022 The Authors. *Ultrasound in Obstetrics & Gynecology* published by John Wiley & Sons Ltd on behalf of International Society of Ultrasound in Obstetrics and Gynecology.

## Abstract 2

This article's abstract has been translated into Spanish and Chinese. Follow the links from the abstract to view the translations.

## RESUMEN

**Ecografía del tercer trimestre para el diagnóstico prenatal del espectro de placenta acreta en mujeres con placenta previa: resultados del estudio ADoPAD**

**Objetivo:**

*Evaluar la eficacia de la ecografía del tercer trimestre para el diagnóstico del trastorno del espectro de la placenta acreta (PAS) clínicamente significativo (PAS, por sus siglas en inglés) en mujeres con placenta baja o placenta previa*.

**Métodos:**

*Este fue un estudio prospectivo multicéntrico de mujeres embarazadas de edad ≥18 años a las que se les diagnosticó con placenta baja (<20mm del orificio cervical interno) o placenta previa (que cubre el orificio cervical interno) en la ecografía de ≥26+0 semanas de gestación, entre octubre de 2014 y enero de 2019. La sospecha ecográfica de PAS se planteó ante la presencia de al menos uno de estos signos en una ecografía en escala de grises: (1) obliteración del espacio hipoecogénico entre el útero y la placenta; (2) interrupción de la interfaz hiperecogénica entre la serosa uterina y la pared vesical; (3) lagunas placentarias anómalas. Los exámenes histopatológicos se realizaron según un protocolo predefinido, en el que los hallazgos ecográficos se ocultaron a los patólogos. Para evaluar la capacidad de la ecografía para detectar el PAS clínicamente significativo, se consideró como estándar de referencia un resultado compuesto que incluía la necesidad de tratamiento activo en el momento del parto y la confirmación histopatológica del PAS. Se consideró que el PAS era clínicamente significativo si, además de la confirmación histológica, se realizó al menos uno de estos procedimientos después del parto: uso de globo intrauterino hemostático, sutura uterina compresiva, histerectomía en el periparto, ligadura de la arteria uterina/hipogástrica o embolización de la arteria uterina. Se evaluó la eficacia para el diagnóstico de cada signo ecográfico para el PAS clínicamente significativo en todas las mujeres y en el subgrupo que tuvo al menos una cesárea previa y placenta anterior. La probabilidad posterior a la prueba se evaluó mediante los nomogramas de Fagan*.

**Resultados:**

*Un total de 568 mujeres se sometieron a ecografías transabdominales y transvaginales durante el periodo de estudio. De ellas, 95 dieron a luz en hospitales locales, por lo que no se dispuso de la patología de la placenta según el protocolo del estudio. Entre las 473 mujeres de las que se disponía de patología placentaria, se diagnosticó PAS clínicamente significativo en 99 (21%), que incluían 36 casos de placenta acreta, 19 de placenta increta y 44 de placenta percreta. La mediana de la edad gestacional en el momento de la evaluación ecográfica fue de 31,4 (rango intercuartil, 28,6–34,4) semanas. Un espacio hipoecogénico normal entre el útero y la placenta redujo la probabilidad posterior a la prueba de PAS clínicamente significativo del 21% al 5% en las mujeres con placenta baja o placenta previa en el tercer trimestre del embarazo y del 62% al 9% en el subgrupo con cesárea previa y placenta anterior. La ausencia de lagunas placentarias redujo la probabilidad posterior a la prueba de PAS clínicamente significativo del 21% al 9% en mujeres con placenta baja o placenta previa en el tercer trimestre del embarazo y del 62% al 36% en el subgrupo con cesárea previa y placenta anterior. Cuando se observaron lagunas placentarias anómalas en la ecografía, la probabilidad posterior a la prueba de PAS clínicamente significativo aumentó del 21% al 59% en toda la cohorte y del 62% al 78% en el subgrupo con cesárea previa y placenta anterior. La interfaz hiperecogénica interrumpida entre la serosa uterina y la pared de la vejiga aumentó la probabilidad posterior a la prueba de un PAS clínicamente significativo del 21% al 85% en mujeres con placenta baja o placenta previa y del 62% al 88% en el subgrupo con cesárea previa y placenta anterior. Cuando los tres marcadores ecográficos estaban presentes, la probabilidad posterior a la prueba de PAS clínicamente significativo aumentó del 21% al 89% en toda la cohorte y del 62% al 92% en el subgrupo con cesárea previa y placenta anterior*.

**Conclusiones:**

*La ecografía en escala de grises tiene una buena eficacia en el diagnóstico para identificar embarazos con bajo riesgo de PAS en una población de alto riesgo de mujeres con placenta baja o placenta previa. La ecografía puede utilizarse con seguridad para guiar las decisiones de tratamiento y concentrar los recursos en las pacientes con mayor riesgo de PAS clínicamente significativo*.

## 摘要

**前置胎盘孕妇的胎盘植入谱系产前诊断的妊娠第三期超声检查:ADoPAD研究结果**

**目的:**

*评价妊娠第三期超声检查对低置或前置胎盘孕妇临床上显著的胎盘植入谱系疾病(PAS)的诊断效果。*

**方法:**

本研究属于一项前瞻性多中心研究，研究对象为2014年10月至2019年1月期间，妊娠≥*26*+*0周时，经超声诊断为低置胎盘(距离宫颈内口小于20毫米)或前置胎盘(覆盖宫颈内口)的孕妇(年龄18周岁或以上)。超声怀疑PAS的条件是灰阶超声至少出现以下一种迹象:(1)子宫和胎盘之间的低回声空间闭塞；(2)子宫浆膜和膀胱壁之间的低回声界面中断；(3)异常胎盘陷窝。根据预先确定的方案进行组织病理学检查，超声检查结果对病理学医师设盲。为了评价超声检查发现临床上显著的PAS的能力，将分娩时需要积极管理和PAS的组织病理学确认在内的综合结果视为参考标准。除了组织病理学确认外，分娩后至少进行以下一种程序，则认为PAS具有临床显著性:使用宫腔止血球囊、子宫压迫缝合术、围产期子宫切除术、子宫/腹下动脉结扎或子宫动脉栓塞术。对所有孕妇以及至少一次剖腹产和前置胎盘的亚组评价每种超声征象对临床上显著的PAS的诊断效果。后验概率使用Fagan列线图进行评价。*

**结果:**

*研究期间共计568名孕妇接受经腹部和经阴道超声检查。 其中95名孕妇在本地医院分娩，因此未能遵照研究方案进行胎盘病理检查。在473名提供胎盘病理学资料的孕妇中，99人(21%)被诊断为患有临床显著PAS，包括36例侵入性胎盘、19例植入性胎盘和44例穿透性胎盘。超声评估时的中位胎龄为31.4(四分位数间距，28.6‐34.4)周。子宫和胎盘之间存在正常的低回声空间，因此妊娠第三期低置或前置胎盘孕妇的临床显著PAS后验概率从21%降至5%，而经历过剖腹产和前置胎盘的亚组则从62%降至9%。在无胎盘陷窝的情况下，妊娠第三期低置或前置胎盘孕妇的临床显著PAS后验概率从21%降至9%，而经历过剖腹产和前置胎盘的亚组则从62%降至36%。若超声检查观察到异常胎盘陷窝，整组的临床显著PAS后验概率从21%增加到59%，而经历过剖腹产和前置胎盘的亚组则从62%增加到78%。若发生子宫浆膜和膀胱壁之间的低回声界面中断，低置或前置胎盘孕妇的临床显著PAS后验概率从21%增加到85%，而经历过剖腹产和前置胎盘的亚组则从62%增加到88%。当所有三种超声征象都存在时，整组的临床显著PAS后验概率从21%增加到89%，而经历过剖腹产和前置胎盘的亚组则从62%增加到92%。*

**结论:**

*在低置或前置胎盘的高危人群中，灰阶超声对识别低风险PAS的孕妇具有良好的诊断效果。 超声可安全地用于指导管理决策并将资源集中在具有较高临床显著PAS风险的患者身上。*

**CONTRIBUTION**

*What are the novel findings of this work?*

This is the first multicenter prospective study showing that grayscale ultrasound has good diagnostic performance to identify pregnancies at low risk of placenta accreta spectrum disorders (PAS) in a high‐risk population of patients with low‐lying placenta or placenta previa. The probability of clinically significant PAS decreased from 21% to 11%, 9% and 5% when the uterine serosa–bladder wall interface was normal, when placental lacunae were absent and when the hypoechogenic retroplacental space was non‐interrupted, respectively.

*What are the clinical implications of this work?*

In patients with low‐lying placenta or placenta previa in the third trimester, ultrasound may be safely used to guide management decisions and concentrate resources on patients with higher risk of clinically significant PAS.

## INTRODUCTION

Placenta accreta spectrum disorder (PAS) is a pregnancy complication that occurs when the chorionic villi invade the myometrium. On the basis of the depth of myometrial invasion, it is classified into placenta accreta, placenta increta and placenta percreta^1^. PAS is associated with increased maternal mortality and morbidity, including uterine rupture before viability, massive hemorrhage, multiorgan failure and the need for hysterectomy^2^. Placenta previa and a history of Cesarean delivery are the main risk factors for PAS, with the risk of placental adhesive disorder increasing with the number of Cesarean sections^3^. Ultrasound is usually considered the first‐line tool in the diagnosis of abnormal placentation. Its diagnostic performance is generally reported to be good, with sensitivity ranging from 77% to 97% and specificity up to 97%^4^. A recent systematic review and meta‐analysis including 3907 pregnancies presenting with placenta previa or low‐lying placenta and one or more prior Cesarean deliveries identified 328 (8.4%) cases of placenta previa accreta, of which 298 (90.9%) were diagnosed prenatally on ultrasound^5^. However, there is wide variation in prenatal detection rates for PAS depending on the ultrasound signs used, operator experience, scanning conditions, equipment used and gestational age. In particular, color Doppler imaging is more susceptible to interoperator variability than is grayscale imaging^6^. Antenatal diagnosis has been shown to reduce maternal morbidity^7^; however, recent population studies have shown that up to two‐thirds of PAS cases remain undiagnosed prenatally^6^. Differences in detection rates between studies may be

attributed to a combination of limited sample size, retrospective design, variability in study inclusion criteria and whether confirmation of diagnosis of PAS is at delivery or by histopathology^6^. As the presence of histopathological features of PAS without complications such as bleeding is of limited clinical significance^8^, we planned a multicenter prospective study to evaluate the diagnostic performance of third‐trimester ultrasound for the diagnosis of clinically significant PAS.

## METHODS

This was a multicenter prospective observational study (ADoPAD, Antenatal Diagnosis of Placental Attachment Disorders) involving 16 Italian hospitals, conducted from October 2014 to January 2019. The study was registered with ClinicalTrials.gov (NCT02442518). The study was approved by the ethics committee of the coordinating center (University of Brescia) and all local ethics committees. Written informed consent was obtained from all study participants. The study was not funded.

We included pregnant women with a low‐lying placenta (< 20 mm from the internal cervical os) or placenta previa (covering the os), aged ≥ 18 years, who underwent ultrasound assessment at ≥ 26 + 0 weeks to assess placental location. All patients underwent a further scan at 34–36 weeks' gestation for repeat assessment of placental location. During the antenatal visits, maternal demographic characteristics and medical and obstetric history were recorded, and ultrasound examination was performed to assess the placenta. Both transabdominal and transvaginal examinations were performed in all cases, using only grayscale ultrasound. First, transabdominal imaging was performed to obtain an overview of placental location and start assessing the regions of concern. Then, a transvaginal scan was performed, inserting the transducer carefully into the vagina up to a short distance from the cervix, under continuous observation of the image. A sagittal scan of the whole length of the cervix and lower part of the uterus was obtained in each woman. The internal cervical os and the lower part of the uterine wall were visualized clearly in all cases, moving the transvaginal probe from side to side. Measurements were obtained by tracing the distance between the lower edge of the placental tissue and the margin of the internal cervical os in the absence of uterine contraction. The thickness of the lower placental edge was measured within 1 cm of the meeting point of the basal and chorionic plates^9^. All ultrasound examinations were performed with a moderately full bladder to allow assessment of the interface between the uterine serosa and the bladder wall.

**Figure 1:**
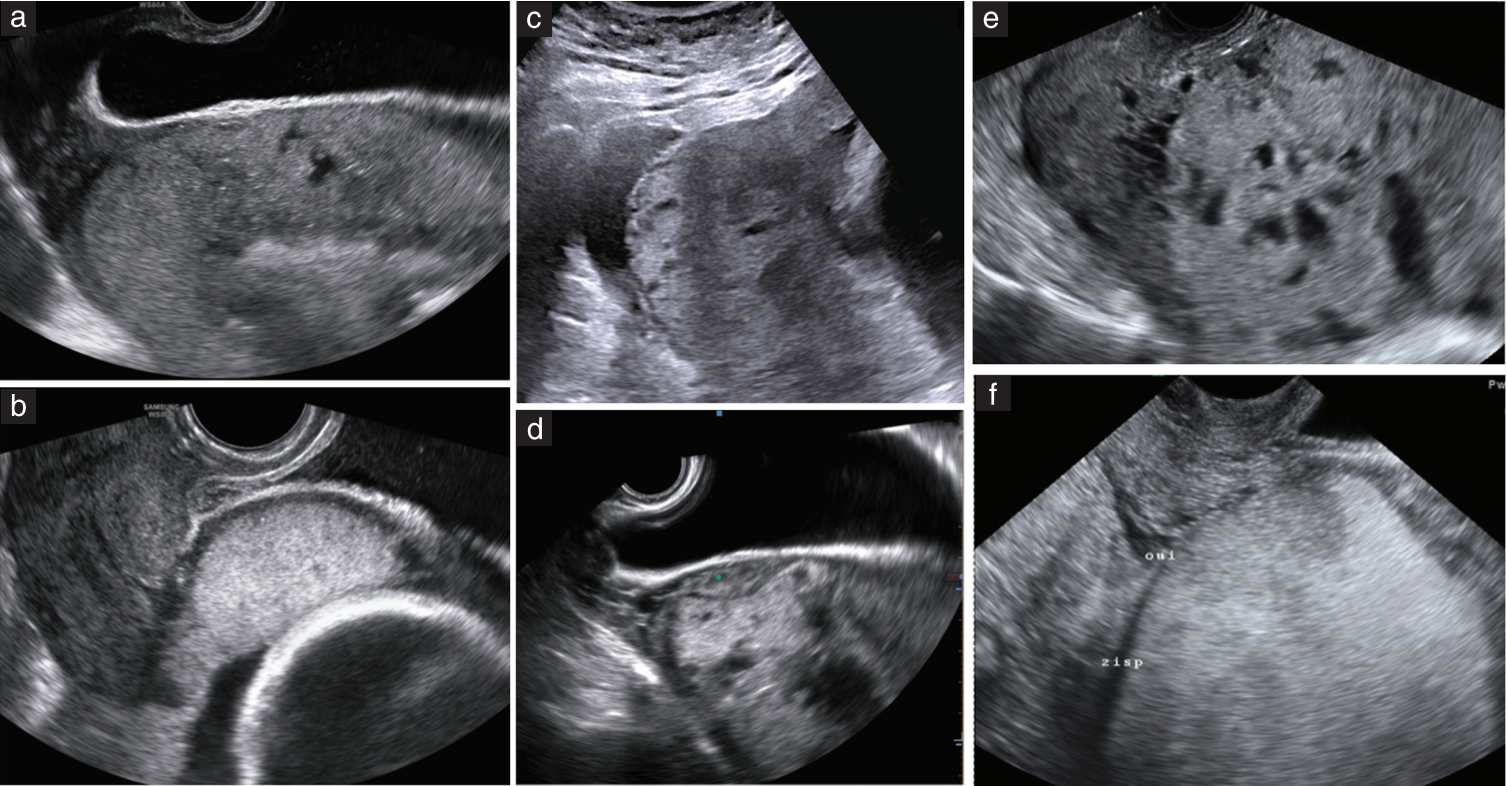
Grayscale ultrasound images obtained at 30–32 weeks' gestation, showing presence (a,c,e) and absence (b,d,f) of sonographic signs of placenta accreta spectrum disorder. (a,b) Obliterated (a) and normal (b) hypoechogenic space between uterus and placenta; (c,d) interrupted (c) and normal (d) hyperechogenic interface between uterine serosa and bladder wall; (e,f) presence (e) and absence (f) of abnormal placental lacunae.

Ultrasound suspicion of PAS was raised in the presence of at least one of these signs: (1) obliteration of the hypoechogenic space between the uterus and the placenta; (2) interruption of the hyperechogenic interface between the uterine serosa and the bladder wall; (3) abnormal placental lacunae, defined as the presence of numerous lacunae including some that are large and irregular (Finberg Grade 3), often containing turbulent flow visible on grayscale imaging^10^. Examples of the presence and absence of these ultrasound signs are shown in Figure 1. Pregnancy outcome, including diagnosis of PAS, was obtained from the hospital records for all patients. The clinicians managing the delivery were aware of the ultrasound findings.

To assess the ability of ultrasound to detect clinically significant PAS, a composite outcome comprising the need for active management at delivery and histopathological confirmation of PAS was considered as reference standard. In the presence of any ultrasound sign of PAS, the placenta was labeled as possibly accreta. Clinical suspicion of PAS at the time of delivery was raised whenever the placenta did not separate at all or only partially at delivery and/or the attempt to remove it led to brisk hemorrhage^8^. Pathological examinations were performed according to a predefined protocol, with the pathologists blinded to the ultrasound findings. Pathological diagnosis of placenta accreta, whether performed on hysterectomy specimens or only on placentas in those in whom the uterus was retained, relied on findings of placental villi in direct apposition to the myometrium in the absence of intermediate decidual layers between anchoring villi and muscular cells^1^, ^8^. PAS was considered of clinical significance if, in addition to histological confirmation, at least one of these procedures was carried out after delivery: use of hemostatic intrauterine balloon, compressive uterine suture, peripartum hysterectomy, uterine/hypogastric artery ligation or uterine artery embolization. When none of these procedures was required to stop bleeding, the histological diagnosis of PAS was not considered clinically significant.

### Statistical analysis

Continuous variables were presented as median and interquartile range (IQR), and comparisons were performed using the Kruskal–Wallis test or Mann–Whitney *U*‐test. Categorical variables were presented as *n* (%), and comparisons were performed using the chi‐square test or Fisher's exact test, as appropriate. *P*‐values < 0.05 were considered significant. Sensitivity, specificity, diagnostic accuracy, positive likelihood ratio, negative likelihood ratio, diagnostic odds ratio, positive predictive value and negative predictive value (NPV) were calculated for each ultrasound sign and their combination, in all women for whom pathology data were available and in the subgroup with at least one previous Cesarean section and anterior placenta. Fagan nomograms were used to estimate how the result of each ultrasound sign changed the probability that a patient had clinically significant PAS^11^. Cases with indeterminate or missing ultrasound data were not used for calculation of diagnostic accuracy.

After a pilot estimate of the prevalence of PAS in our population as 0.2 (unpubl. data), we estimated that, for a predetermined value of sensitivity of 80% and α = 0.05, with precision of estimate set at 10%, specificity of 95% and an expected dropout rate of 10%, a sample size of 348 would be needed. Statistical analysis was performed using Stata version 13.1 (StataCorp, College Station, TX, USA). The results of this study are reported in accordance with the Standards for Reporting Diagnostic Accuracy (STARD) statement^12^.

## RESULTS

**Figure 2:**
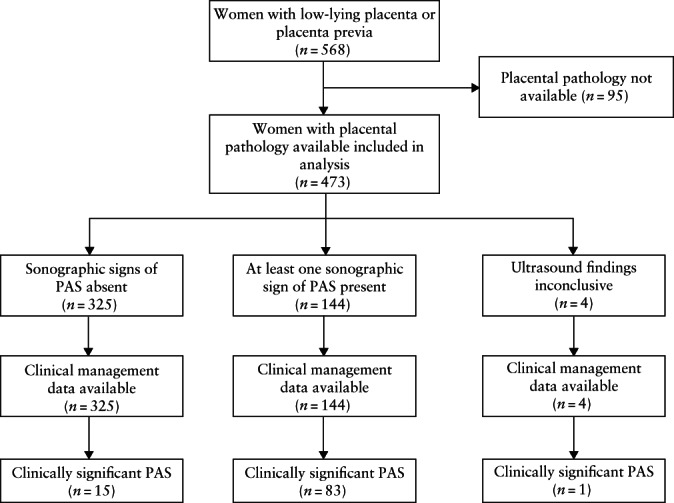
STARD flowchart showing inclusion in study of women with low‐lying placenta or placenta previa, ultrasound findings related to placenta accreta spectrum disorder (PAS) and final diagnosis of clinically significant PAS, which was defined as histological confirmation of PAS in addition to need for active management at delivery.

A total of 568 women with low‐lying placenta or placenta previa were included in the study. Of these, 95 delivered in local hospitals, and placental pathology according to the study protocol was therefore not available. The frequency of ultrasound signs of PAS and active management in the study population are shown in Table S1. Among the 473 women for whom placental pathology was available, clinically significant PAS was diagnosed in 99 women (prevalence, 20.9% (95% CI, 17.0–24.9%)), comprising 36 cases of placenta accreta, 19 cases of placenta increta and 44 cases of placenta percreta. In 16 of the 36 cases of superficial PAS, the diagnosis was performed after hysterectomy; in the remaining 20 cases, pathological diagnosis of superficial placenta accreta was performed on the placentas. In all cases of placenta increta and placenta percreta, the diagnosis was performed after hysterectomy. The STARD diagram showing the flow of participants through the study is shown in Figure 2.

Characteristics and detailed clinical outcomes of the study cohort, according to their history of Cesarean section, are presented in Table 1. At the time of delivery, 368 women had placenta previa and 105 had low‐lying placenta. There were 101 women with one previous Cesarean section, 47 with two, 14 with three and four with four. A posterior placenta was observed in 258/473 (54.5%) women, of whom 21/258 (8.1%) had clinically significant PAS. Sixty‐three women with posterior placenta had at least one previous Cesarean section, of whom 13 had clinically significant PAS. Of the 21 women with clinically significant PAS and posterior placenta, six had a history of dilatation and curettage and two had conceived by *in‐vitro* fertilization. There were 215/473 (45.5%) women with anterior placenta, of whom 78/215 (36.3%) had clinically significant PAS. Of the women with anterior placenta, 103 had at least one previous Cesarean section, of whom 64 had clinically significant PAS. Of the 78 women with clinically significant PAS and anterior placenta, 31 had a history of dilatation and curettage and four conceived by *in‐vitro* fertilization. The median gestational age at the time of ultrasound assessment was 31.4 (IQR, 28.6–34.4) weeks. All patients underwent an additional scan at 34–36 weeks' gestation to reassess placental location. In nine of the 473 cases, the distance between the lower placental edge and the internal cervical os was ≥ 20 mm; three of these had clinically significant PAS and underwent hysterectomy. Table 2 shows the clinical characteristics and clinical outcomes of the study cohort according to the mode of management at delivery.

**Table 1:** Demographic and delivery characteristics, sonographic findings, management at delivery and histopathological diagnosis of 473 women with low‐lying placenta or placenta previa, according to history of previous Cesarean section (CS)

Parameter	Previous CS (*n* = 166)	No previous CS (*n* = 307)	*P*
Age at delivery (years)	35.5 (32.6–38.5)	35.5 (31.9–39.5)	0.691
Prepregnancy body mass index (kg/m^2^)	24.3 (20.7–27.1)	22.7 (20.6–25.0)	0.0049
Caucasian ethnicity	133 (80.1)	262 (85.3)	0.144
Conceived by *in‐vitro* fertilization	5 (3.0)	49 (16.0)	< 0.001
History of dilatation and curettage	64 (38.6)	106 (34.5)	0.384
History of myomectomy	7 (4.2)	31 (10.1)	0.025
GA at initial ultrasound (weeks)	31.0 (28.6–33.9)	31.7 (28.7–34.4)	0.1553
Placental position			< 0.001
Anterior	103 (62.0)	112 (36.5)	
Posterior	63 (38.0)	195 (63.5)	
Cervical length (mm)	37.0 (30.0–40.0)	38.0 (32.0–42.9)	0.073
Placental thickness (mm)	31.4 (18.0–43.0)	21.0 (11.0–31.0)	< 0.0001
Hypoechogenic retroplacental space			< 0.001
Normal	67 (40.4)	281 (91.5)	
Interrupted	97 (58.4)	24 (7.8)	
Not determinable	2 (1.2)	2 (0.7)	
Hyperechogenic uterus–bladder interface			< 0.001
Normal	104 (62.7)	303 (98.7)	
Interrupted	62 (37.3)	4 (1.3)	
Abnormal placental lacunae	84 (50.6)	32 (10.4)	< 0.001
GA at delivery (weeks)	35.6 (34.0–36.7)	36.3 (35.0–37.1)	0.0001
Delivery by CS	162 (97.6)	297 (96.7)	0.779
Placenta covering internal cervical os at time of delivery	134 (80.7)	234 (76.2)	0.261
Birth weight (g)	2540 (2180–2800)	2665 (2340–3000)	0.0012
Blood transfusion	57 (34.3)	52 (16.9)	< 0.001
Units of packed red cells transfused	4 (2–6)	2 (2–4)	0.0077
Intrauterine balloon tamponade	34 (20.5)	75 (24.4)	0.33
Uterine compression sutures	9 (5.4)	10 (3.3)	0.253
Hysterectomy	73 (44.0)	9 (2.9)	< 0.001
Ligation of pelvic vessels	10 (6.0)	2 (0.7)	0.001
Embolization of pelvic vessels	10 (6.0)	3 (1.0)	0.002
Active management	107 (64.5)	87 (28.3)	< 0.001
PAS diagnosis by histopathology	91 (54.8)	63 (20.5)	< 0.001
Clinically significant PAS	77 (46.4)	22 (7.2)	< 0.001
Maternal death	0 (0)	0 (0)	—

Data are given as median (interquartile range) or *n* (%).

GA, gestational age; PAS, placenta accreta spectrum disorder.

**Table 2:** Sonographic findings in third trimester and histopathological diagnosis of 473 women with low‐lying placenta or placenta previa, according to mode of management at delivery

		Active management		
Parameter	No active management (*n* = 279)	Without hysterectomy (*n* = 112)	With hysterectomy (*n* = 82)	*P* *
Placenta previa	202 (72.4)	90 (80.4)	76 (92.7)	< 0.001
Previous Cesarean section	59 (21.1)	34 (30.4)	73 (89.0)	< 0.001
Cervical length (mm)	38.0 (33.0–42.3)	37.2 (30.0–43.0)	35.0 (30.0–40.0)	0.0393
Placental thickness (mm)	20.8 (12.0–30.0)	24.7 (14.4–35.5)	37.2 (23.0–45.0)	0.0001
Hypoechogenic retroplacental space				< 0.001
Normal	251 (90.0)	90 (80.4)	7 (8.5)	
Interrupted	25 (9.0)	22 (19.6)	74 (90.2)	
Not determinable	3 (1.1)	0 (0)	1 (1.2)	
Hyperechogenic uterus–bladder interface				< 0.001
Normal	272 (97.5)	104 (92.9)	31 (37.8)	
Interrupted	7 (2.5)	8 (7.1)	51 (62.2)	
Abnormal placental lacunae	29 (10.4)	27 (24.1)	60 (73.2)	< 0.001
Blood transfusion	23 (8.2)	37 (33.0)	49 (59.8)	< 0.001
Units of packed red cells transfused	2 (2–2)	2 (2–3)	5 (4–8)	0.0001
PAS diagnosis at histopathology	55 (19.7)	20 (17.9)	79 (96.3)	< 0.001
Clinically significant PAS	0 (0)	20 (17.9)	79 (96.3)	< 0.001
Maternal death	0 (0)	0 (0)	0 (0)	—

Data are given as *n* (%) or median (interquartile range).

* *P*‐value represents difference between all three management groups.

PAS, placenta accreta spectrum disorder.

**Figure 3:**
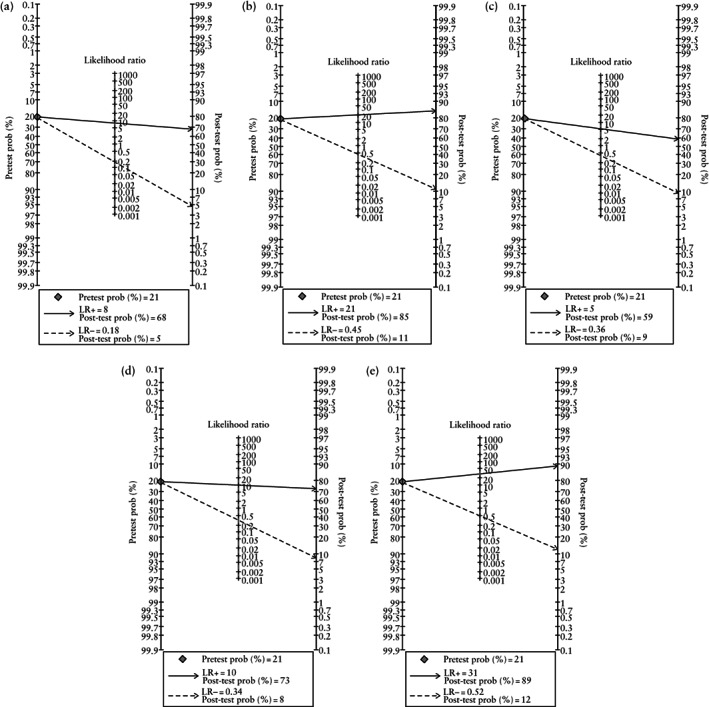
Fagan nomograms showing post‐test probability of clinically significant placenta accreta spectrum disorder for interrupted hypoechogenic retroplacental space (a), interrupted hyperechogenic uterus–bladder interface (b), presence of abnormal placental lacunae (c), interrupted hypoechogenic retroplacental space in addition to abnormal placental lacunae (d) and presence of all three markers (e) in 473 women with low‐lying placenta or placenta previa. In four cases, the operator was not able to assess the retroplacental hypoechogenic space. LR+, positive likelihood ratio; LR−, negative likelihood ratio; prob, probability.

The diagnostic performance of the three ultrasound markers (interrupted hypoechogenic retroplacental space, interrupted hyperechogenic uterus–bladder interface and presence of abnormal placental lacunae) and their combination for the antenatal diagnosis of clinically significant PAS in all women with available placental pathology is shown in Table 3 and Table S2. The diagnostic accuracy of grayscale third‐trimester ultrasound for clinically significant PAS was 76.9%, 77.9% and 86.6% in the presence of an interrupted uterine serosa–bladder wall interface, abnormal placental lacunae and interrupted hypoechogenic retroplacental space, respectively. The diagnostic performance of the three ultrasound markers and their combination for prediction of clinically significant PAS in the subgroup of women with previous Cesarean section and anterior placenta (*n* = 103) is shown in Table S3. The post‐test probabilities of clinically significant PAS in all women with available placental pathology and in those with previous Cesarean section and anterior placenta are shown in Figures 3 and S1, respectively.

**Table 3:** Diagnostic performance of third‐trimester ultrasound markers for antenatal diagnosis of clinically significant placenta accreta spectrum disorder in 473 women with low‐lying placenta or placenta previa

Ultrasound marker	Sensitivity (%)	Specificity (%)	Accuracy (%)	LR+	LR−	DOR	PPV (%)	NPV (%)
Interrupted hypoechogenic retroplacental space	83.7 (74.4–90.4)	89.5 (85.9–92.4)	86.6 (82.6–90.6)	7.96 (5.84–10.85)	0.18 (0.12–0.29)	43.6 (23.3–81.5)	67.8 (58.7–78.6)	95.4 (92.6–96.3)
Interrupted hyperechogenic uterus–bladder interface	56.6 (46.2–66.5)	97.3 (95.1–98.7)	76.9 (72.0–81.9)	21.2 (11.2–39.9)	0.45 (0.36–0.56)	47.4 (22.8–98.5)	84.8 (73.9–92.5)	89.4 (86.0–92.2)
Abnormal placental lacunae	68.7 (58.6–77.6)	87.2 (83.3–90.4)	77.9 (73.0–82.9)	5.35 (3.98–7.19)	0.36 (0.27–0.48)	14.9 (8.85–25.1)	58.6 (49.1–67.7)	91.3 (87.9–94.0)
Abnormal placental lacunae + interrupted hypoechogenic retroplacental space	68.4 (58.2–77.4)	93.3 (90.2–95.6)	80.8 (76.0–85.6)	10.2 (6.79–15.2)	0.34 (0.25–0.45)	29.9 (16.7–53.7)	72.8 (62.6–81.6)	91.8 (88.5–95.3)
All three markers	50.0 (39.7–60.3)	98.4 (96.5–99.4)	74.2 (69.2–79.2)	30.9 (13.6–70.1)	0.52 (0.42–0.62)	60.8 (25.3–146)	89.1 (77.8–95.9)	88.2 (84.7–91.1)

Values in parentheses are 95% CI.

Hypoechogenic retroplacental space could not be assessed in four cases.

DOR, diagnostic odds ratio; LR+, positive likelihood ratio; LR−, negative likelihood ratio; NPV, negative predictive value; PPV, positive predictive value.

## DISCUSSION

We have shown that grayscale ultrasound findings in the third trimester have a good NPV for clinically significant PAS in a high‐risk population. Normal hypoechogenic space between the uterus and the placenta reduced the post‐test probability of PAS from 21% to 5% in women with low‐lying placenta or placenta previa in the third trimester (Figure 3a) and from 62% to 9% in the subgroup of women with previous Cesarean section and anterior placenta (Figure S1a). The absence of placental lacunae reduced the post‐test probability of PAS from 21% to 9% in the whole cohort (Figure 3c) and from 62% to 36% in the subgroup with previous Cesarean section and anterior placenta (Figure S1c). On the other hand, in women with low‐lying placenta or placenta previa, the post‐test probability for clinically significant PAS increased from 21% to 59% in the presence of abnormal placental lacunae in the third trimester (Figure 3c) and to 85% when an interrupted hyperechogenic interface between the uterine serosa and bladder wall was observed (Figure 3b). In the subgroup of women with previous Cesarean section and anterior placenta, the post‐test probability for clinically significant PAS increased from 62% to 78% in the presence of abnormal placental lacunae (Figure S1c) and to 88% when the hyperechogenic uterus–bladder interface was interrupted (Figure S1b). When all three markers were present, the post‐test probability for clinically significant PAS increased from 21% to 89% in the whole cohort (Figure 3e) and from 62% to 92% in the subgroup with previous Cesarean section and anterior placenta (Figure S1e). Although interrupted hypoechogenic space and lacunae have been associated with an increased likelihood of invasive placentation, they may be present even in women with placenta previa without myometrial invasion^13^. Our results are in agreement with those of Pilloni *et al*.^14^, who found, in a prospective single‐center study, that loss or irregularity of the retroplacental clear zone had the highest sensitivity for PAS (81%). In addition, Calì *et al*. found that the most effective ultrasound criteria for detection of morbidly adherent placenta were loss or irregularity of the clear space between the placenta and uterus and hypervascularity of the uterine serosa–bladder wall interface, with NPVs of 96.7% and 97%, respectively^15^. These data compare well with the NPV of 95.4% for PAS that we obtained when a normal retroplacental hypoechogenic space was seen on grayscale ultrasound.

The main strengths of the present study are its prospective design, the use of a standardized approach to collect and evaluate ultrasound markers of PAS, and its multicenter nature, which are likely to increase the external validity of our findings. A limitation of this study is its potentially low generalizability, as we evaluated a high‐risk population in the third trimester of pregnancy, with a high overall prevalence of PAS (21%), particularly among women with anterior placenta and previous Cesarean section (62%). Another limitation is that a standardized pathological examination of the placenta was not available in 16.7% of the initial cohort. These women delivered in their local hospital after assessment in one of the referral centers participating in the study, and had more favorable outcomes than women included in the analysis, with no hysterectomy. This patient selection process may explain the higher prevalence of PAS in our cohort of women with placenta previa or low‐lying placenta compared to the 11.1% reported by Jauniaux *et al*. in a recent meta‐analysis of population‐based studies^16^. Another possible explanation is the different definition of placenta accreta used in our study. Jauniaux *et al*.^16^ used a clinical grading based on surgical findings at delivery for the diagnosis of accreta placentation^17^ and histopathological findings when a Cesarean hysterectomy was performed, i.e. placental villi directly attached to the myometrium without interposing decidua or invading the uterine wall. As there are no objective criteria for the intraoperative diagnosis of PAS, and our clinical aim was the prediction of major morbidity regardless of whether invasive placentation was confirmed on histopathology or at surgery, we chose to use the term ‘clinically significant PAS’, which was defined as the finding of placental villi in direct apposition to the myometrium in the absence of intermediate decidual layers between anchoring villi and muscular cells, together with significant bleeding, even without hysterectomy, in order to include also milder or focal forms of histologically diagnosed PAS that nonetheless caused significant maternal hemorrhagic morbidity^8^.

Although color Doppler had the best predictive accuracy for PAS in a systematic review and meta‐analysis including 3707 patients at risk for invasive placentation^4^, we did not evaluate abnormal color Doppler findings in our study as these are subjective and there are currently no quantitative indicators to measure increased vascularity in routine practice. The location of the placenta in the lower uterine segment alone is sufficient to increase the vascularity in comparison to cases with a fundal placenta, and, in women with prior Cesarean delivery without PAS, increased vascularity is often seen in the scarred lower uterine segment–urinary bladder interface. Indeed, the amount of vascularity may be influenced by the ultrasound machine settings used, even in cases in which the placenta is not implanted in the lower uterine segment. In almost all publications involving placenta accreta, only women with known risk factors, such as low anterior placenta and previous Cesarean delivery, were examined^8^. Therefore, selection bias may explain the increased vascularity at the lower uterine segment–urinary bladder interface. Whether such increased vascularity is the result of abnormal invasion or simply low‐anterior placental location, and how this may be measured objectively, remains to be determined^8^.

In conclusion, a low anterior placenta with invasive placentation poses great challenges for peripartum management. Such pregnancies are most likely to experience complications, and therefore prenatal diagnosis is likely to have the greatest impact. This multicenter prospective study showed that grayscale ultrasound has good ability to identify pregnancies at low risk of PAS in this otherwise high‐risk population. Ultrasound may be used safely to guide management decisions, concentrating resources on patients with the higher risk of clinically significant PAS.

## Supporting information


**Figure S1** Fagan nomograms showing post‐test probability of clinically significant placenta accreta spectrum disorder for interrupted hypoechogenic retroplacental space (a), interrupted hyperechogenic uterus–bladder interface (b), presence of abnormal placental lacunae (c), interrupted hypoechogenic retroplacental space in addition to abnormal placental lacunae (d), and presence of all three markers (e) in 103 women with low‐lying placenta or placenta previa who had at least one previous Cesarean section and anterior placenta. In one case, the operator was not able to assess the retroplacental hypoechogenic space.

**Table S1** Ultrasound findings and management at delivery in the whole study population, according to whether placental pathology was available
**Table S2** 2 × 2 tables for diagnostic performance of third‐trimester ultrasound markers for antenatal diagnosis of clinically significant placenta accreta spectrum disorder (PAS) in 473 women with low‐lying placenta or placenta previa
**Table S3** Diagnostic performance of third‐trimester ultrasound markers for antenatal diagnosis of clinically significant placenta accreta spectrum disorder (PAS) in 103 women with low‐lying placenta or placenta previa who had at least one previous Cesarean section and anterior placenta

## Data Availability

The data that support the findings of this study are available from the corresponding author upon reasonable request.
