# Supplementary figures and images for: Third‐trimester ultrasound for antenatal diagnosis of placenta accreta spectrum in women with placenta previa: results from the ADoPAD study
Source: Ultrasound Obstet Gynecol. 2022 Sep 1;60(3):381–9. doi: 10.1002/uog.24889 (PMC9544821; doi:10.1002/uog.24889)

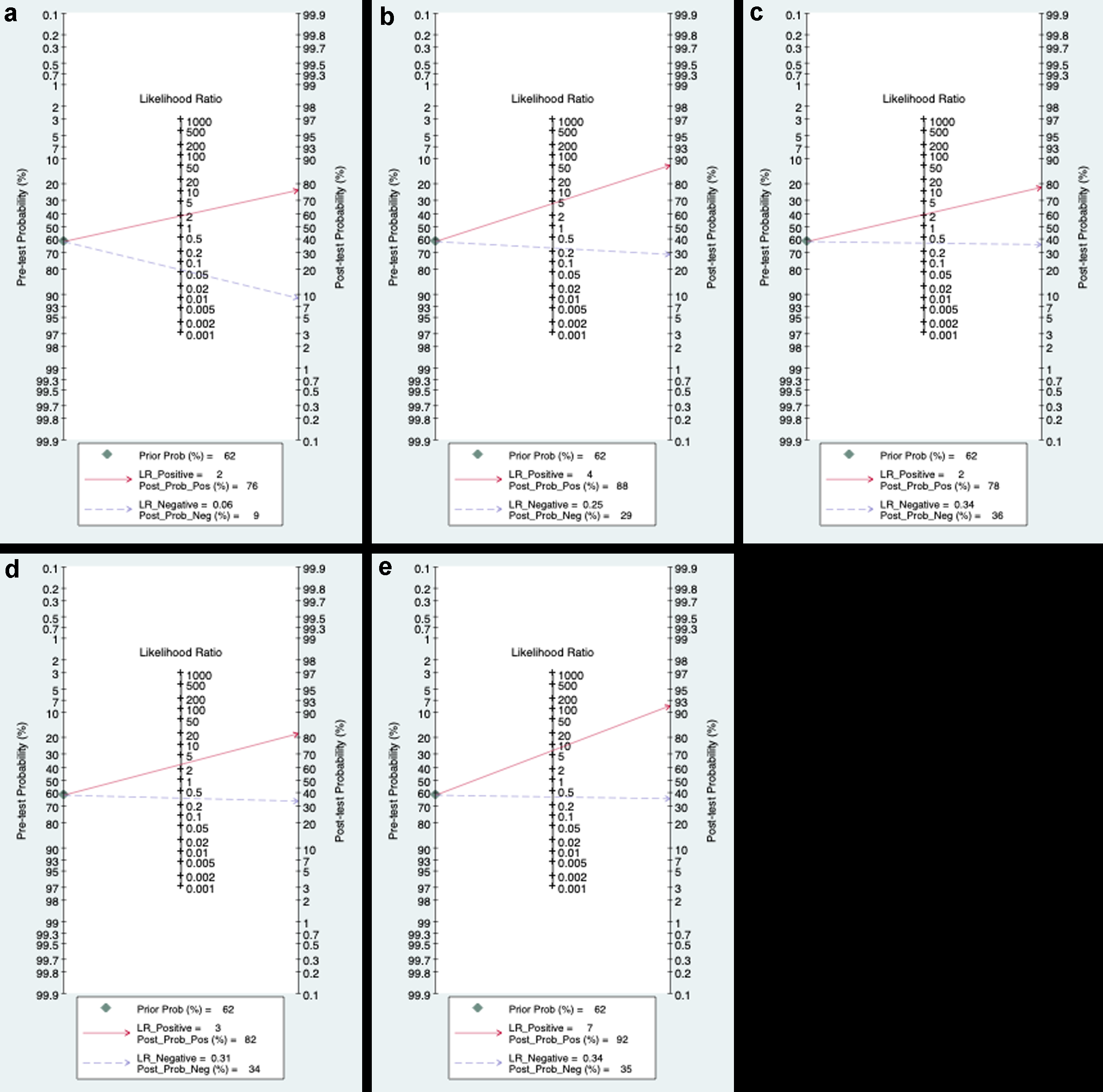

Supplement: Supplementary file 1 — Figure S1 Fagan nomograms showing post‐test probability of clinically significant placenta accreta spectrum disorder for interrupted hypoechogenic retroplacental space (a), interrupted hyperechogenic uterus–bladder interface (b), presence of abnormal placental lacunae (c), interrupted hypoechogenic retroplacental space in addition to abnormal placental lacunae (d), and presence of all three markers (e) in 103 women with low‐lying placenta or placenta previa who had at least one previous Cesarean section and anterior placenta. In one case, the operator was not able to assess the retroplacental hypoechogenic space. [file UOG-60-381-s002.tif]
