# Supplementary material for: Third‐trimester ultrasound for antenatal diagnosis of placenta accreta spectrum in women with placenta previa: results from the ADoPAD study
Source: Ultrasound Obstet Gynecol. 2022 Sep 1;60(3):381–9. doi: 10.1002/uog.24889 (PMC9544821; doi:10.1002/uog.24889)
Supplement: Supplementary file 2 — Table S1 Ultrasound findings and management at delivery in the whole study population, according to whether placental pathology was available Table S2 2 × 2 tables for diagnostic performance of third‐trimester ultrasound markers for antenatal diagnosis of clinically significant placenta accreta spectrum disorder (PAS) in 473 women with low‐lying placenta or placenta previa Table S3 Diagnostic performance of third‐trimester ultrasound markers for antenatal diagnosis of clinically significant placenta accreta spectrum disorder (PAS) in 103 women with low‐lying placenta or placenta previa who had at least one previous Cesarean section and anterior placenta [file UOG-60-381-s001.docx]

**Table S1** Ultrasound findings and management at delivery in the whole study population, according to whether placental pathology was available

| **Parameter** | | **Placental pathology unavailable (n=95)** | **Placental pathology available (n=473)** | **p** |
| --- | --- | --- | --- | --- |
| Placenta Covering the internal cervical os | | 60 (63%) | 368 (78%) | 0.003 |
| Cervical length (mm) | | 39.9 (from 34.5 to 44.5) | 37.5 (from 32 to 42) | 0.007 |
| Placental thickness (mm) | | 17.4 (from 10.3 to 32) | 13.2 (from 5 to 23.5) | 0.0193 |
| Hypoechogenic retroplacental space | |  |  | <0.0001 |
| Normal | 88 (92.6%) | 348 (73.5%) |  |  |
| Interrupted | 6 (6.3%) | 121 (25.5%) |  |  |
| Not determinable | 1 (1.1%) | 4 (1%) |  |  |
| Hyperechogenic uterus–bladder interface | |  |  | 0.008 |
| Normal | 91 (95.8%) | 407 (86%) |  |  |
| Interrupted | 4 (4.2%) | 66 (14%) |  |  |
| Abnormal placental lacunae | |  |  | <0.0001 |
| Absent | 89 (93.7%) | 357 (75.5%) |  |  |
| Present | 6 (6.3%) | 116 (24.5%) |  |  |
| Blood transfusion | | 11 (11.5%) | 109 (23%) | 0.012 |
| Units of packed red cells transfused | | 2 (from 1 to 4) | 3 (from 2 to 5) | 0.0924 |
| Intrauterine balloon tamponade | | 11 (11.5%) | 109 (23%) | 0.012 |
| Uterine compression sutures | | 2 (2.1%) | 19 (4%) | 0.553 |
| Hysterectomy | | 0 | 82 (17.3%) | <0.0001 |
| Pelvic vessels ligation | | 1 (1%) | 12 (2.5%) | 0.705 |
| Pelvic vessels embolization | | 1 (1%) | 13 (2.7%) | 0.484 |
| Active management | | 14 (14.7%) | 194 (41%) | <0.0001 |
| PAS at pathology | | NA | 154 (32,5%) | NA |
| Clinically significant PAS | | NA | 99 (21%) | NA |
| Maternal death | | 0 | 0 |  |

Data are reported as number (n) and percentage (%) or as median and interquartile range (IQR). PAS, placenta accreta spectrum disorder

**Table S2** 2 × 2 tables for diagnostic performance of third-trimester ultrasound markers for antenatal diagnosis of clinically significant placenta accreta spectrum disorder (PAS) in 473 women with low-lying placenta or placenta previa

|  | **Obliterated hypoechogenic space** | **Normal hypoechogenic space** | Total |
| --- | --- | --- | --- |
| **PAS** | 82 | 16 | 98 |
| **Non PAS** | 39 | 332 | 371 |
| Total | 121 | 348 | 469 |

|  | **Interrupted hyperechogenic uterus–bladder interface** | **Normal hyperechogenic uterus–bladder interface** | Total |
| --- | --- | --- | --- |
| **PAS** | 56 | 43 | 99 |
| **Non PAS** | 10 | 364 | 374 |
| Total | 66 | 407 | 473 |

|  | **Abnormal placental lacunae** | **No abnormal lacunae** | Total |
| --- | --- | --- | --- |
| **PAS** | 68 | 31 | 99 |
| **Non PAS** | 48 | 326 | 374 |
| Total | 116 | 357 | 473 |

|  | **Abnormal placental lacunae and obliterated hypoechogenic space** | **No abnormal lacunae and normal hypoechogenic space** | Total |
| --- | --- | --- | --- |
| **PAS** | 67 | 31 | 98 |
| **Non PAS** | 25 | 346 | 371 |
| Total | 92 | 377 | 469 |

|  | **All 3 markers abnormal** | **No all 3 markers abnormal** | Total |
| --- | --- | --- | --- |
| **PAS** | 49 | 49 | 98 |
| **Non PAS** | 6 | 365 | 371 |
| Total | 55 | 414 | 469 |

**Table S3** Diagnostic performance of third-trimester ultrasound markers for antenatal diagnosis of clinically significant placenta accreta spectrum disorder (PAS) in 103 women with low-lying placenta or placenta previa who had at least one previous Cesarean section and anterior placenta

|  | **Obliterated hypoechogenic space** | **Normal hypoechogenic space** | Total |
| --- | --- | --- | --- |
| **PAS** | 61 | 2 | 63 |
| **Non PAS** | 19 | 10 | 29 |
| Total* | 80 | 22 | 102 |

|  | **Interrupted hyperechogenic uterus–bladder interface** | **Normal hyperechogenic uterus–bladder interface** | Total |
| --- | --- | --- | --- |
| **PAS** | 51 | 13 | 64 |
| **Non PAS** | 7 | 32 | 39 |
| Total | 58 | 45 | 103 |

|  | **Abnormal placental lacunae** | **No abnormal lacunae** | Total |
| --- | --- | --- | --- |
| **PAS** | 50 | 14 | 64 |
| **Non PAS** | 14 | 25 | 39 |
| Total | 64 | 39 | 103 |

|  | **Abnormal placental lacunae and Obliterated hypoechogenic space** | **No abnormal lacunae and normal hypoechogenic space** | Total |
| --- | --- | --- | --- |
| **PAS** | 49 | 14 | 63 |
| **Non PAS** | 11 | 28 | 39 |
| Total* | 60 | 42 | 102 |

|  | **All 3 markers abnormal** | **No all 3 markers abnormal** | Total |
| --- | --- | --- | --- |
| **PAS** | 44 | 19 | 63 |
| **Non PAS** | 4 | 35 | 39 |
| Total* | 48 | 54 | 102 |

| **Ultrasound marker** | **Prevalence**  **(%)** | **Sensitivity (%)** | **Specificity (%)** | **Diagnostic accuracy (%)** | **LR+** | **LR-** | **DOR** | **PPV (%)** | **NPV (%)** |
| --- | --- | --- | --- | --- | --- | --- | --- | --- | --- |
| Interrupted hypoechogenic retroplacental space* | 62% (52%-71.2%) | 96.8% (89%-99.6%) | 51.3% (34.8%-67.6%) | 0.741 (0.658-0.823) | 1.99 (1.44-2.75) | 0.0619 (0.0153-0.25) | 32.1 (6.86-150.67) | 76.3% (65.4%-85.1%) | 90.9% (70.8%-98.9%) |
| Interrupted hyperechogenic uterus–bladder interface | 62% (52%-71.5%) | 79.7% (67.8%-88.7%) | 82.1% (66.5%-92.5%) | 0.809 (0.73-0.887) | 4.44 (2.24-8.78) | 0.248 (0.149-0.411) | 17.9 (6.56-48.9) | 87.9% (76.7%-95%) | 71.1% (55.7%-83.6%) |
| Abnormal placental lacunae | 62% (52%-71.5%) | 78.1% (66%-87.5%) | 64.1% (47.2%-78.8%) | 0.711 (0.619-0.803) | 2.18 (1.4-3.38) | 0.341 (0.203-0.574) | 6.38 (2.66-15.3) | 78.1% (66%-87.5%) | 64.1% (47.2%-78.8%) |
| Abnormal placental lacunae + interrupted hypoechogenic retroplacental space* | 62% (52%-71.2%) | 77.8% (65.5%-87.3%) | 71.8% (55.1%-85%) | 0.748 (0.66-0.836) | 2.76 (1.64-4.63) | 0.31 (0.187-0.511) | 8.91 (3.59-22.1) | 81.7% (69.6%-90.5%) | 66.7% (50.5%-80.4%) |
| All three markers* | 62% (52%-71.2%) | 69.8% (57%-80.8%) | 89.7% (75.8%-97.1%) | 0.798 (0.723-0.873) | 6.81 (2.65-17.5) | 0.336 (0.227-0.497) | 20.3 (6.52-62.2) | 91.7% (80%-97.7%) | 64.8% (50.6%-77.3%) |

Values in parentheses are 95% CI. *Retroplacental hypoechogenic space could not be assessed in one case. DOR, diagnostic odds ratio; LR+, positive likelihood ratio; LR- negative likelihood ratio; NPV, negative predictive value; PPV, positive predictive value, PAS, placenta accreta spectrum disorder.
